# Supplementary material for: SyNDI: synchronous network data integration framework
Source: BMC Bioinformatics. 2018 Nov 6;19:403. doi: 10.1186/s12859-018-2426-5 (PMC6219086; doi:10.1186/s12859-018-2426-5)
Supplement: Supplementary file 5 — Files of the example presented in Additional file 4. This additional file is a zip package that contains all files used in Additional file 4. Detailed descriptions of each of these files are presented in a README file included in this zip package. (GZ 30411 kb) [file 12859_2018_2426_MOESM5_ESM.gz › SyNDITools/Meme2Fimo/meme_4.9.0_4.tar.gz!/meme_4.9.0/website/html/general-faq.html]

GENERAL - FAQ


|  |  |
| --- | --- |
|  | MEME Suite The MEME Suite web application requires the use of JavaScript   Javascript doesn't seem to be available on your browser.  Frequently Asked Questions     ---    - **Q.**   I haven't received my MEME confirmation email? - **Q.**   Why don't the motif occurrences in the "Summary of Motifs" block diagrams match the occurrences shown in   the individual motif block diagrams? - **Q.**   MAST returns "Your sequence contains unrecognized characters..." - **Q.**   Is it possible to introduce specific amino acid   compositions in the MEME and MAST programs? - **Q.**   I don't really understand what the pseudocount   parameters for tomtom represent, nor how to use them   properly. - **Q.**   MEME Suite won't compile!   I get error messages about libxml2 or libxslt.   What should I do?  ---    - **Q.**   I haven't received my MEME confirmation email? **A.** You should have received a   confirmation email that your job is submitted. If you   haven't received this email, then you might have typed   in the wrong email address.  Alternatively, your mail program may be filtering the   MEME emails as spam, so check your folders designated   as junk e-mail or spam. Try to configure your mail   program not to filter out emails from   meme@nbcr.net.  Please also check with your mail server administrator   and see whether your email has been filtered out by   spam filters run automatically on all incoming   messages by your organization.  ---    - **Q.** Why don't the motif occurrences in the   "Summary of Motifs" block diagrams match the   occurrences shown in the individual motif block   diagrams? **A.** The motif occurrences shown for a motif in   its section of the output are the ones discovered by   the MEME algorithm. These occurrences are then used   to create a position specific scoring matrix (PSSM)   for the motif. The PSSMs for all of the motifs   discovered by MEME are then used to search for   non-overlapping sets of occurrences of motifs with   p-values under 0.0001 using the MAST algorithm. These   are displayed in the "Summary of Motifs" block   diagram. Individual motif occurrences will sometimes   be missing from the "Summary of Motifs" block diagram   because of the non-overlapping or minimum p-value   requirements. Conversely, there may be motif   occurrences in the summary diagram that were not   discovered by the MEME algorithm.  ---  - **Q.** MAST returns "Your sequence contains   unrecognized characters..." **A.** The file must be a plain text file. MSWord   files, for example, are not acceptable. MSWord files   can be converted to plain text by:     "File"-->"Save As"-->"Save As Type"-->"Plain Text'   (\*.txt)".  ---  - **Q.** Is it possible to introduce specific amino   acid compositions in the MEME and MAST programs? **A.** MEME can use higher-order background models.   However, doing this requires a locally installed MEME   and running "fasta-get-markov", which is included in   the distribution. The relevant switch to MEME is   "-bfile".  MAST can only use zero-order (compositional)   models. This is done automatically on the web server   when searching the "upstream" databases using a file   based on all upstream regions for the particular   organism. As with MEME, users of locally installed   MAST can specify their own background files. The   switch to MAST is "-bfile".  ---  - **Q.**   I don't really understand what the pseudocount   parameters for tomtom represent, nor how to use them   properly. **A.** Essentially, a pseudocount corresponds to a   Bayesian prior. If you flip a coin 10 times and   observe 10 heads in a row, then without a prior, you   would estimate the probability of observing a heads on   the next round as 100%. But because you have a prior   belief that the coin is probably fair, you might   pretend that you started off with one heads and one   tails. Then you would say that you've seen 1 tails   and 11 heads, so your probability of heads would be   91.7%. If you keep flipping the coin all day, and you   only ever see heads, then the estimated probability of   heads would eventually get very close to 100%.  The motif pseudocounts work they same way: they allow   you to say that, even though we've never seen an "A"   in position 3 of the motif, it's still a possibility.   Pseudocounts are important for motifs with few known   occurrences. If you have a motif built from 100   instances, then the pseudocount is not likely to be   important, but if the motif is based on only a handful   of instances, then it is.  Motifs coming out of MEME already have pseudocounts   included in them. Otherwise, it's a good idea to add   a small pseudocount.  ---  - **Q.**   MEME Suite won't compile!   I get error messages about `libxml2` or `libxslt`.   What should I do? **A.**   `libxml2`   is a library for parsing XML files.   `libxslt`   is a library for processsing XML files.   using XSLT stylesheets.   MEME Suite will try to use the versions of these libraries   already installed on your system.   If the libraries can't be found on your system,   MEME Suite will build its own copies from source included in the   MEME Suite distribution.   If MEME Suite detects a local copy of `libxml2`,   but no local copy of `libxslt`, it will try to build   its own copy of `libxslt`, but it may run into   conflicts between the installed versions of `libxml2`   and included version of `libxslt` that will keep MEME suite   from compiling.   If you run into this problem you can force MEME Suite to build   both libraries from the included source.   Just add the options `--enable-build-libxml2` and   `--enable-build-libxslt` to your `configure`   command line.   For further information about `configure` options see   the installation guide.  --- |
